# Comparison between the Acute Pulmonary Vascular Effects of Oxygen with Nitric Oxide and Sildenafil

**DOI:** 10.3389/fped.2015.00016

**Published:** 2015-03-03

**Authors:** Ronald W. Day

**Affiliations:** ^1^Division of Pediatric Cardiology, Primary Children’s Hospital, University of Utah, Salt Lake City, UT, USA

**Keywords:** nitric oxide, oxygen, phosphodiesterase V inhibitor, pulmonary arterial hypertension

## Abstract

**Objective:** Right heart catheterization is performed in patients with pulmonary arterial hypertension to determine the severity of disease and their pulmonary vascular reactivity. The acute pulmonary vascular effect of inhaled nitric oxide is frequently used to identify patients who will respond favorably to long-term vasodilator therapy. This study sought to determine whether the acute pulmonary vascular effects of oxygen with nitric oxide and intravenous sildenafil are similar.

**Methods:** A retrospective, descriptive study of 13 individuals with pulmonary hypertension who underwent heart catheterization and acute vasodilator testing was performed. The hemodynamic measurements during five phases (21–53% oxygen, 100% oxygen, 100% oxygen with 20 ppm nitric oxide, 21–51% oxygen, and 21–51% oxygen with 0.05–0.29 mg/kg intravenous sildenafil) of the procedures were compared using analysis of variance. A linear regression analysis and a Bland Altman plot were used to compare the percent change in mean pulmonary arterial pressure and the percent change in pulmonary vascular resistance from baseline with oxygen and nitric oxide, and from baseline with sildenafil.

**Results:** Mean pulmonary arterial pressure and pulmonary vascular resistance acutely decreased with 100% oxygen with nitric oxide and 21–51% oxygen with sildenafil. Pulmonary blood flow during sildenafil was greater than pulmonary blood flow during 100% oxygen and 100% oxygen with nitric oxide. The pH, right atrial pressure, and left atrial pressure did not change during the five phase of heart catheterization. Mean pulmonary arterial pressure (millimeter of mercury, mean ± standard error of the mean) was 38 ± 4 during 21–53% oxygen, 32 ± 3 during 100% oxygen, 29 ± 2 during 100% oxygen with nitric oxide, 37 ± 3 during 21–51% oxygen, and 32 ± 2 during 21–51% oxygen with sildenafil. There was not a significant correlation between the percent change in pulmonary vascular resistance from baseline with oxygen and nitric oxide, and from baseline with sildenafil (*r*^2^ = 0.011, *p* = 0.738). The Bland Altman analysis demonstrated statistical agreement between the effects of oxygen with nitric oxide and sildenafil. However, differences were large enough to limit the interchangeable use of these vasodilators in a clinical setting.

**Conclusion:** Oxygen with nitric oxide and sildenafil decreased pulmonary vascular resistance. However, the pulmonary vascular effects of oxygen and nitric oxide do not reliably predict the acute response to sildenafil. Additional studies are needed to determine whether the acute response to sildenafil can be used to predict the long-term response to treatment with an oral phosphodiesterase V inhibitor.

Pulmonary hypertension is a serious disease associated with constriction, cellular hypertrophy and proliferation, inflammation, and *in situ* thrombosis of the small vessels of the pulmonary circulation (1). Patients often respond more favorably to long-term treatment if their pulmonary vascular resistance decreases during acute vasodilator testing during heart catheterization (2–4). However, the response to short acting agents may not predict the long-term response to medications that act on different pathways of signal transduction. The acute hemodynamic effects of oxygen with nitric oxide and diltiazem were not similar in a previous study of patients with pulmonary hypertension (5).

Sildenafil is a phosphodiesterase V inhibitor that increases the 6-min walk performance of adults with pulmonary arterial hypertension (6). Sildenafil also improved 6-min walk performance and hemodynamic measurements of children with pulmonary hypertension (7). However, low-dose sildenafil failed to improve exercise performance short-term, and high-dose sildenafil was potentially associated with a decrease in long-term survival in a randomized, placebo-controlled study of children with pulmonary arterial hypertension (8). These studies did not report whether patients with a favorable response to acute vasodilator testing had a better response to sildenafil long-term. At Primary Children’s Hospital, patients with pulmonary hypertension typically undergo an evaluation of pulmonary vascular reactivity with supplemental oxygen, inhaled nitric oxide, and at least one additional agent during heart catheterization. Nitric oxide and sildenafil decrease pulmonary vascular resistance through a common pathway. This study sought to compare the acute effects of inhaled nitric and the acute effects of intravenous sildenafil in patients with pulmonary hypertension.

## Methods

The Institutional Review Board of the University of Utah approved this retrospective study. The records of patients who underwent acute pulmonary vasodilator testing with supplemental oxygen, inhaled nitric oxide, and intravenous sildenafil were reviewed. At Primary Children’s Hospital, hemodynamic measurements are performed while patients are breathing enough supplemental oxygen to achieve an oxygen saturation greater than 87%, 100% oxygen, 100% oxygen with 20 parts per million (ppm) nitric oxide, 21–100% oxygen, and 21–100% oxygen with intravenous diltiazem, intravenous sildenafil, or intravenous epoprostenol. The severity of disease and the acute response to oxygen with nitric oxide are used to determine whether to evaluate the response to diltiazem, sildenafil, or epoprostenol. Patients with mild to moderate pulmonary hypertension or a reasonably good response to oxygen with nitric oxide are usually evaluated with 21–30% oxygen and diltiazem or sildenafil. Patients with moderate to severe pulmonary hypertension and a limited response to oxygen with nitric oxide are usually evaluated with 100% oxygen and epoprostenol. In this study, an estimate of 100% oxygen was administered at a flow rate of 15 L/min with a non-rebreathing facemask (one patient) or an inspired fraction of approximately 1.0 through an endotracheal tube. The dose of nitric oxide was held constant at 20 ppm during each study because the pulmonary vasodilatory effects of 12 and 60 ppm nitric oxide were similar in a previous study (9). Nitric oxide was administered with oxygen because the vasodilatory effects of these agents may be additive (3, 9–11). The dose of intravenous sildenafil has been somewhat arbitrary, beginning with a dose of 0.05–0.15 mg/kg followed by additional doses until a change in systemic blood pressure is observed. The mean dose of intravenous sildenafil was 0.15 mg/kg with a median of 0.16 mg/kg and a range of 0.05–0.29 mg/kg. Cardiac index was calculated by the Fick principle using estimated values of oxygen consumption between 120 and 160 L/min-m^2^ based primarily upon age, body surface area, and heart rate (12, 13). A consistent estimate of oxygen consumption was used for all stages of each evaluation of pulmonary vascular reactivity. Patients were not given background therapy on the day of the procedure.

### Inclusion criteria

Thirteen patients who were evaluated with intravenous sildenafil during heart catheterization were included in this study. Patients with a pulmonary hypertension classification in Group 1 or Group 3 were included in the study (14). All patients had a baseline mean pulmonary arterial pressure greater than 25 mm Hg with a median of 33 mm Hg and a range of 26–70 mm Hg and a pulmonary vascular resistance greater than 3 units-m^2^ during initial baseline measurements.

### Exclusion criteria

Patients with a pulmonary hypertension classification in Group 2, Group 4, or Group 5 were not included in the study. Patients with a functional single ventricle were excluded from this study.

### Acutely responsive patients

A favorable acute response to oxygen with nitric oxide or sildenafil was defined with the following criteria:

- a 20% decrease in mean pulmonary arterial pressure from baseline measurements (1),
- a 20% decrease in pulmonary vascular resistance from baseline measurements,
- a decrease in mean pulmonary arterial pressure ≥10 mm Hg to a value of <35 mm Hg for patients with a baseline mean pulmonary arterial pressure ≥35 mm Hg, or a decrease in mean pulmonary arterial pressure to a value of <25 mm Hg for patients with a baseline mean pulmonary arterial pressure <35 mm Hg.

Each criterion was considered because it is unknown whether one is superior to the other, particularly for patients who have a baseline mean pulmonary arterial pressure less than 40 mm Hg (3).

### Statistical analysis

Analysis of variance for repeated measures was used to identify significant differences (*p* < 0.05) between inhaled nitric oxide with supplemental oxygen and intravenous sildenafil for arterial blood gases, heart rate, blood flow, vascular resistance, and blood pressure. A linear regression analysis was used to identify potential linear correlations between the pulmonary vasodilatory effects of oxygen, oxygen with nitric oxide, and sildenafil. Numerical data are expressed as mean ± standard error of the mean. A Bland Altman analysis was performed to compare the percent decrease in pulmonary vascular resistance achieved with an acute trial of oxygen with nitric oxide and an acute trial of intravenous sildenafil.

## Results

### Demographic information

There were 13 patients (7 female). The median age of patients was 12 years with a range of 0.5–35 years. The diagnostic classification of patients is listed in Table 1. Three of the patients with congenital heart disease and one of the patients with lung disease have Down syndrome. Heart catheterization was performed when a diagnosis of pulmonary hypertension was initially confirmed in seven patients, and for follow-up evaluations of pulmonary vascular reactivity in six patients. No patient had more than one evaluation of pulmonary vascular reactivity included in this study. Patients reside at altitudes of 1300–2000 m. Heart catheterization was performed at an altitude of approximately 1500 m. An anesthesiologist provided sedation for all patients. Assisted ventilation was used to electively support 12 patients during heart catheterization. There were no complications associated with the procedures.

**Table 1:** **Diagnostic classification of patients**.

	Number of patients
Pulmonary arterial hypertension
Idiopathic	1
Associated with
Congenital heart disease (repaired)	2
Congenital heart disease (not repaired)	3
Partial anomalous pulmonary venous return	1
Atrial septal defect	2
Connective tissue disease	2
Lung disease	5

The median amount of supplemental oxygen during baseline 1 was 23% with a range of 21–53%. The median amount of supplemental oxygen during baseline 2 and the sildenafil phase was 23% with a range of 21–51%.

### Pulmonary vascular reactivity

**Figure 1:**
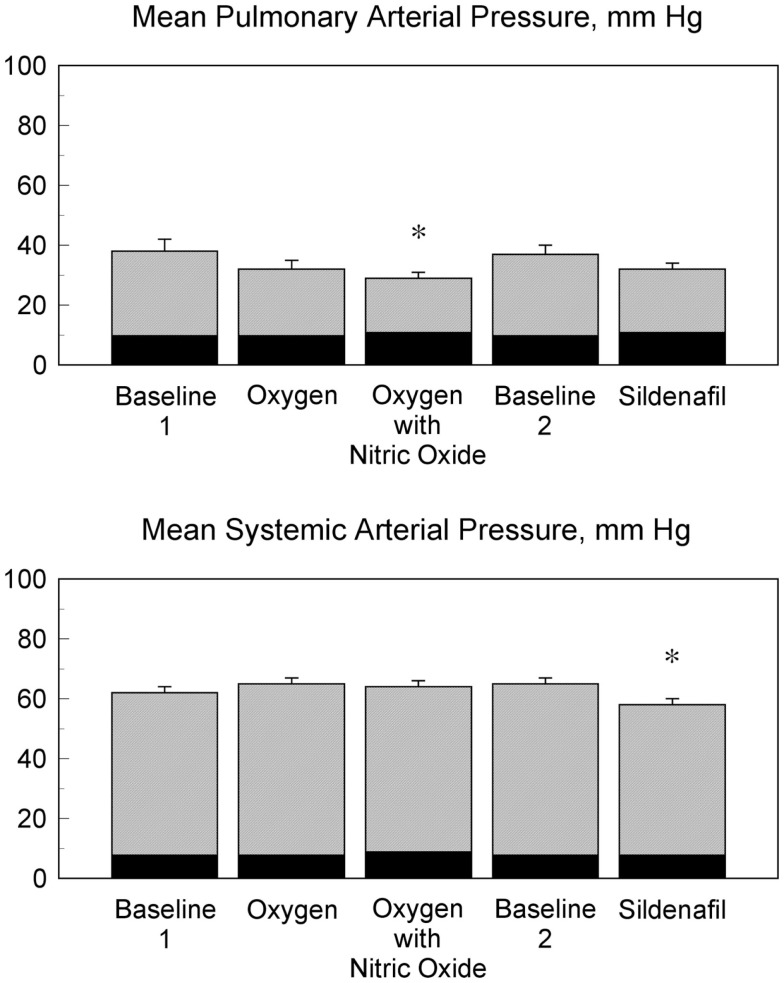
**Influence of vasodilatory agents on pulmonary and systemic arterial pressures**. The mean pulmonary arterial pressures and the mean systemic arterial pressures for 13 patients during five phases of heart catheterization are shown. The mean pulmonary arterial pressure was lower during oxygen with nitric oxide and baseline 1 (*p < 0.05). The mean systemic arterial pressure during treatment with an intravenous infusion of sildenafil was lower than the mean systemic arterial pressure during oxygen, oxygen with nitric oxide, and baseline 2 (*p < 0.05). The corresponding mean pulmonary arterial wedge pressures are shown in black with the mean pulmonary arterial pressures. The corresponding right atrial pressures are shown in black with the mean systemic arterial pressures.

The results of arterial blood gas, heart rate, blood flow, and vascular resistance measurements are shown in Table 2. The mean pulmonary arterial blood pressures, mean pulmonary arterial wedge pressures, mean systemic arterial blood pressures, and mean right atrial pressures during each stage of the hemodynamic evaluations are shown in Figure 1. Oxygen with nitric oxide and sildenafil both decreased pulmonary arterial pressure and pulmonary vascular resistance in comparison to the preceding baseline measurements. Sildenafil decreased systemic arterial pressure and systemic vascular resistance. The use of supplemental oxygen was associated with a mild decrease in heart rate.

**Table 2:** **Arterial blood gases, heart rate, blood flow, and vascular resistance**.

Phase	pH	PO_2_, mm Hg	HR, min^−1^	*Q*_p_, L/min-m^2^	*R*_p_, units-m^2^	*R*_s_, units-m^2^
Baseline 1	7.41 ± 0.02	62 ± 4	82 ± 6	3.53 ± 0.33	8.7 ± 1.5	17.7 ± 1.4
100% Oxygen	7.42 ± 0.01	255 ± 32^a^	75 ± 6	3.14 ± 0.25	7.7 ± 1.3	20.1 ± 1.3
100% Oxygen with Nitric Oxide	7.42 ± 0.02	287 ± 31^a^^,^^b^	75 ± 6^b^	3.25 ± 0.27^b^	6.1 ± 1.0^a^	19.4 ± 1.2^b^
Baseline 2	7.43 ± 0.02	64 ± 3	81 ± 6	3.48 ± 0.29	8.4 ± 1.4	18.6 ± 1.3
Sildenafil	7.41 ± 0.01	58 ± 3	83 ± 6	4.00 ± 0.38	5.7 ± 0.8^a^	14.0 ± 0.9^a^

*n = 13, mean ± SEM*.

*HR, heart rate; PO_2_, arterial oxygen tension; Qp, pulmonary blood flow; *R*_p_, pulmonary vascular resistance index; *R*_s_, systemic vascular resistance index*.

*^a^Significant difference in comparison to the preceding baseline measurement, *p* < 0.05*.

*^b^Significant difference between measurements for oxygen with nitric oxide and sildenafil, *p* < 0.05*.

**Figure 2:**
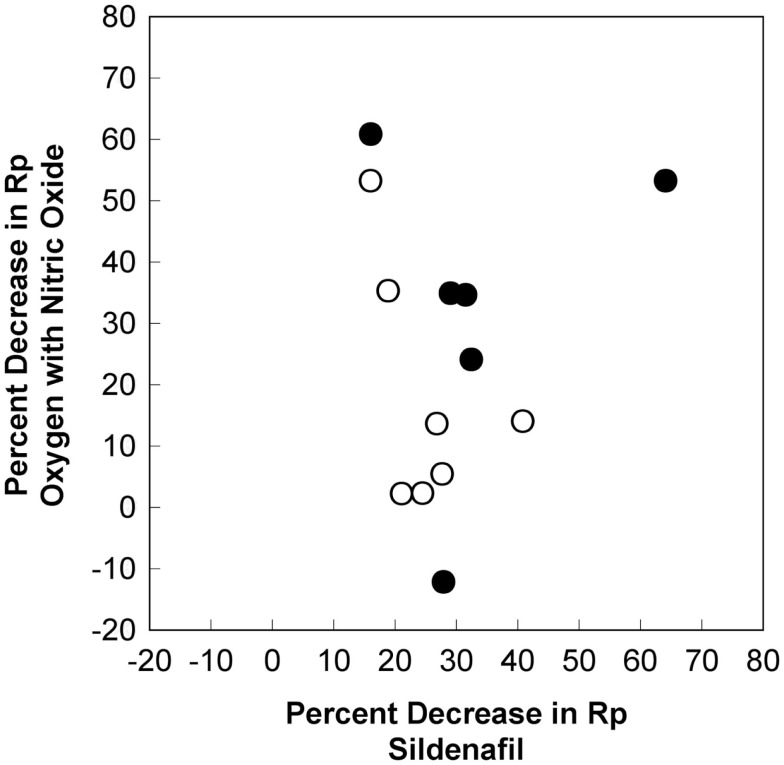
**Differences in the pulmonary vasodilatory effects for oxygen with nitric oxide and sildenafil**. The percent decrease in pulmonary vascular resistance with oxygen and nitric oxide and the percent decrease in pulmonary vascular resistance with sildenafil for each patient are shown. There is no linear correlation for the pulmonary vasodilatory effects of these agents (*r*^2^ = 0.011, *p* = 0.738). There is no correlation for patients with an initial baseline mean pulmonary arterial pressure ≥35 mm Hg (dark circles) or for patients with an initial baseline mean pulmonary arterial pressure <35 mm Hg (light circles). *R*_p_, pulmonary vascular resistance index.

There was a significant correlation between the values of pulmonary vascular resistance (*r*^2^ = 0.861, *p* < 0.001) and mean pulmonary arterial pressure (*r*^2^ = 0.800, *p* < 0.001) during baseline 1 and baseline 2. There was a significant correlation between the values of pulmonary vascular resistance (*r*^2^ = 0.869, *p* < 0.001) and mean pulmonary arterial pressure (*r*^2^ = 0.680, *p* < 0.001) during oxygen and sildenafil. There was a significant correlation between the values of pulmonary vascular resistance (*r*^2^ = 0.782, *p* < 0.001) and mean pulmonary arterial pressure (*r*^2^ = 0.662, *p* < 0.001) during oxygen with nitric oxide and sildenafil. Despite these significant correlations, there was not a significant correlation between the percent change in pulmonary vascular resistance (*r*^2^ = 0.011, *p* = 0.738) and the percent change in mean pulmonary arterial pressure (*r*^2^ = 0.170, *p* = 0.161) between baseline 1 and oxygen with nitric oxide and baseline 2 and sildenafil. The percent decrease in pulmonary vascular resistance with oxygen and nitric oxide and the percent decrease in pulmonary vascular resistance with sildenafil for each patient are shown in Figure 2.

**Figure 3:**
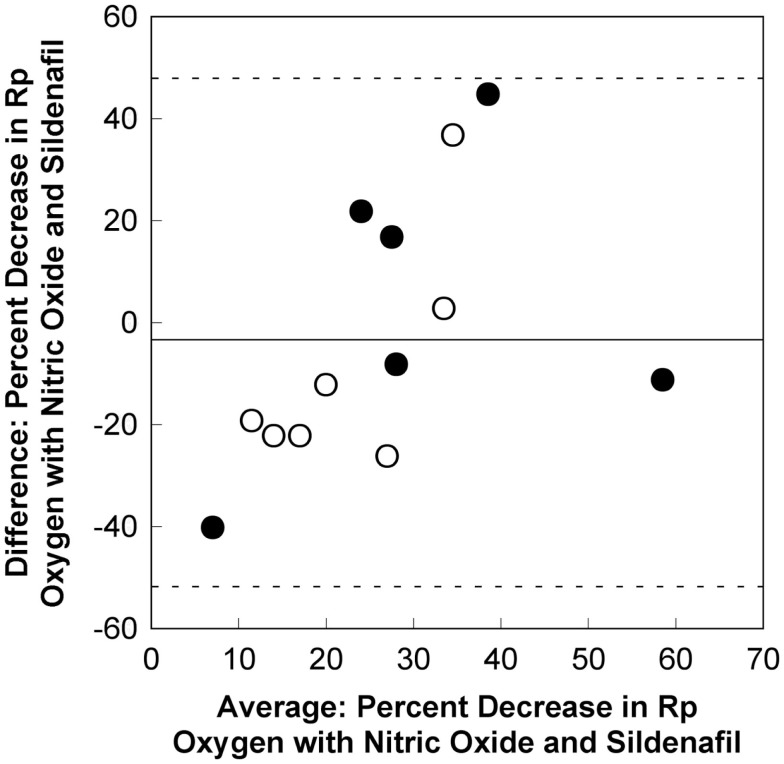
**A Bland Altman plot for the comparison between differences in the percent decrease in pulmonary vascular resistance for oxygen with nitric oxide and sildenafil**. There is statistical evidence of agreement between the pulmonary vasodilatory effects for oxygen with nitric oxide and sildenafil. The mean difference between the percent decrease in pulmonary vascular resistance for oxygen with nitric oxide and sildenafil is −2.8 (solid line). All values fall within the 95% confidence intervals (±1.96 standard deviations, dashed lines). However, the difference between the percent decrease for oxygen with nitric oxide and sildenafil exceeds 20% for 7 of the 13 patients. This appears to be true for patients with an initial baseline mean pulmonary arterial pressure ≥35 mm Hg (dark circles) and for patients with an initial baseline mean pulmonary arterial pressure <35 mm Hg (light circles). *R*_p_, pulmonary vascular resistance index.

The plot for the Bland Altman analysis is shown in Figure 3. All of the values for the differences in percent decrease in pulmonary vascular resistance fall within the 95% confidence intervals (±1.96 standard deviations). This suggests that there is statistical agreement between the results of acute vasodilator testing with oxygen with nitric oxide and sildenafil. Unfortunately, the difference for the percent decrease in pulmonary vascular resistance for the two tests exceeds 20% for 7 of the 13 patients. Plots for comparisons in differences between the decrease mean pulmonary artery pressure and percent decrease in mean pulmonary artery pressure also showed statistical agreement between the results of acute vasodilator testing with oxygen with nitric oxide and sildenafil. The difference in the decrease in mean pulmonary arterial pressure exceeded 5 mm Hg in 4 of the 13 patients. The difference in the percent decrease in mean pulmonary arterial pressure exceeded 20% in 3 of the 13 patients.

The number of responders using each criterion for a favorable acute response to oxygen with nitric oxide or sildenafil is shown in Table 3. More responders to sildenafil were identified if a 20% decrease in pulmonary vascular resistance was used as a criterion because sildenafil seemed to increase pulmonary blood flow. There were 7 patients who experienced a 20% decrease in pulmonary vascular resistance with oxygen and nitric oxide and 10 patients who experienced a 20% decrease in pulmonary vascular resistance with sildenafil. However, only four patients had a 20% decrease in pulmonary vascular resistance with both medications.

**Table 3:** **Number of responsive patients**.

	Oxygen with nitric oxide	Sildenafil
20% decrease in MPAP	6	1
20% decrease in *R*_p_	7	10
MPAP ≥35 mm Hg, *n* = 6
10 mm Hg decrease in MPAP to a value <35 mm Hg	2	0
MPAP <35 mm Hg, *n* = 7
Decrease in MPAP <25 mm Hg	4	1

*MPAP, mean pulmonary arterial pressure; *R*_p_, pulmonary vascular resistance index*.

Patients were treated with the following agents long-term: one patient with supplemental oxygen alone; two patients with a calcium channel blocker; six patients with a phosphodiesterase V inhibitor; one patient with an endothelin receptor antagonist; one patient with a calcium channel blocker and a phosphodiesterase V inhibitor; and two patients with a phosphodiesterase V inhibitor, an endothelin receptor antagonist, and a prostacyclin analog.

## Discussion

This study describes the acute pulmonary vascular effects of supplemental oxygen, oxygen with inhaled nitric oxide, and intravenous sildenafil for a subset of patients who underwent heart catheterization for pulmonary arterial hypertension at a children’s hospital. An adequate dose of sildenafil was used to significantly decrease systemic vascular resistance. Oxygen with nitric oxide and sildenafil both decreased pulmonary vascular resistance. However, the magnitude of the response to these agents differed for individual patients. These results suggest that the response to oxygen with nitric oxide and the response to sildenafil may not be sufficiently consistent to use the tests interchangeably in a clinical setting.

Nitric oxide and sildenafil decrease pulmonary vascular resistance through a common pathway. It is unclear why some patients respond more favorably to one agent. Nitric oxide stimulates soluble guanylate cyclase to produce cyclic-GMP. It is possible that sildenafil is less effective in patients with severe endothelial dysfunction and a limited amount of endogenous nitric oxide production. The effects of inhaled nitric oxide and sildenafil may also be limited by abnormalities in other aspects of this pathway of signal transduction (15).

Heart catheterization can safely be used to evaluate the hemodynamic effects of more than one agent. This allows more information to be obtained concerning the vasodilatory potential of individual patients. The United States Food and Drug administration has warned concerning the use of sildenafil in young patients. It may be helpful to use the acute effects of sildenafil when considering the potential risks and benefits of treatment with an oral phosphodiesterase V inhibitor. It seems reasonable to select an agent for long-term therapy if it acutely decreases pulmonary vascular resistance. However, additional studies will be needed to determine whether a favorable or unfavorable acute vasodilatory response reliably predicts the outcome of long-term therapy. Most clinical trials have failed to report whether the results of acute vasodilator testing corresponded with the outcome of treatment. This study was too small to evaluate whether there was a correlation between the acute response to oxygen, nitric oxide, or sildenafil and the long-term efficacy of treatment with a phosphodiesterase V inhibitor. There were only four patients who were treated long-term with a phosphodiesterase V inhibitor alone.

Some of the definitions related to the hemodynamic evaluation of patients with pulmonary hypertension need additional consideration. Patients with a pulmonary vascular resistance of 3 units-m^2^ typically have a mean pulmonary arterial pressure less than 25 mm Hg unless the left atrial pressure is elevated. A decrease in mean pulmonary arterial pressure of 10 mm Hg to a value less than 40 mm Hg may be an appropriate method for acute vasodilator testing in patients with moderate to severe pulmonary arterial pressure. However, it is not useful for patients with a mean pulmonary arterial pressure less than 35 mm Hg if a mean pulmonary arterial pressure of 25 mm Hg might be considered normal. Many patients with a mean pulmonary arterial pressure near 25 mm Hg have a pulmonary vascular resistance greater than 5 units-m^2^. It may be more appropriate to identify acute responders to vasodilators by a 20% decrease in resistance or pressure instead of a mere decrease in mean pressure below 25 mm Hg. For this reason, several definitions for acute reactivity were considered in this study. The clinical application of information from acute vasodilator testing also deserves additional consideration. For example, if a patient with a baseline mean pulmonary arterial pressure of 50 mm Hg experienced a 15 mm Hg decrease with nitric oxide, a 5 mm Hg decrease with diltiazem, and a 12 mm Hg decrease with sildenafil; would it be appropriate to start long-term therapy with a calcium channel blocker alone? Patients with this profile of pulmonary vascular reactivity exist, particularly in a pediatric setting. The mortality and morbidity of pulmonary hypertension is related to the patient’s pulmonary arterial pressure and heart function. It is important to identify agents that decrease pulmonary arterial pressure in our patients at the onset, and throughout the course, of therapy.

### Limitations

This is a retrospective descriptive study with the following limitations:

- A relatively small number of patients were included in the study. Only one patient has idiopathic pulmonary arterial hypertension. Even though the results of acute vasodilator testing have seemingly been influenced more by the severity of disease than the cause of disease at Primary Children’s Hospital. The results of this study may not be relevant for all causes of pulmonary vascular disease.
- This study only included patients who had some response to supplemental oxygen with nitric oxide. It did not include patients with severe pulmonary hypertension who had no response because they were typically evaluated with intravenous epoprostenol instead of sildenafil. It is unknown whether a subgroup of patients may be responsive to sildenafil, yet fail to respond oxygen with nitric oxide.
- It is unknown whether a maximal pulmonary vasodilatory response occurred with the amounts of oxygen, nitric oxide, and sildenafil that were used during acute vasodilator testing in this study. However, a sufficient amount of sildenafil was used to significantly decrease the mean systemic arterial pressure and systemic vascular resistance.
- Levels of cyclic-GMP were not measured to determine whether the effects of oxygen, nitric oxide, and sildenafil correlated with this messenger of signal transduction.
- There were potential errors in the calculations of blood flow and vascular resistance using assumed and consistent values of oxygen consumption. For this reason, the effects of each agent on direct measurements of mean pulmonary arterial pressure and mean systemic pressure were also reported. Three patients had an atrial level shunt that could have influenced the pulmonary arterial pressure.
- It is possible that sildenafil would have decreased the mean pulmonary arterial pressure or pulmonary vascular resistance more with 100% oxygen than with 21–51% oxygen. A lower amount of oxygen was used with sildenafil in order to avoid obscuring a response to sildenafil by the effects of oxygen.
- This study was performed at a mildly increased altitude of 1500 m. The patients also reside at a mildly to moderately increased altitude. Results may be different if hemodynamic measurements are performed at a different altitude.

In conclusion, supplemental oxygen with inhaled nitric oxide and sildenafil both acutely decrease pulmonary vascular resistance in some patients with pulmonary hypertension. Sildenafil also acutely increases pulmonary blood flow and decreases systemic vascular resistance. The magnitudes of the pulmonary vasodilatory effects of oxygen with nitric oxide and sildenafil were different in individual patients. This discrepancy suggests that acute vasodilator testing with oxygen and nitric oxide should not be used as a surrogate to predict the pulmonary vasodilatory effect of a phosphodiesterase V inhibitor.

## Conflict of Interest Statement

Ronald W. Day has no conflict of interest to disclose concerning this study. The Guest Associate Editor, Prof. Maurice Beghetti, declares that despite having collaborated with the author, Dr. Ronald W. Day, the review process was handled objectively and no conflict of interest exists.
